# Detection of pesticide residue distribution on fruit surfaces using surface-enhanced Raman spectroscopy imaging

**DOI:** 10.1039/c7ra11927e

**Published:** 2018-01-25

**Authors:** Jiannan Chen, Daming Dong, Song Ye

**Affiliations:** Beijing Key Laboratory of Digital Plant, National Engineering Research Center for Information Technology in Agriculture, Beijing Academy of Agriculture and Forestry Sciences Beijing 100097 China damingdong@hotmail.com +86-10-51503411; School of Electronic Engineering and Automation, Guilin University of Electronic Technology Guilin 541000 China

## Abstract

Surface-enhanced Raman spectroscopy (SERS) is an emerging technique for the detection of pesticide residues on food surfaces, permitting quantitative measurement of pesticide residues without pretreating the sample. However, previous studies have mainly involved the single Raman spectrum of samples, while have given little information on pesticide residue distribution. In this paper, gold nanoparticles were used as surface enhancers to obtain the Raman spectra of omethoate and chlorpyrifos, using the Raman shifts of 413 cm^−1^ (omethoate) and 346 & 634 cm^−1^ (chlorpyrifos) as the peaks of interest. Different concentrations of pesticide solution were quantitatively analyzed and the regression curve model was established, whereby the solutions of omethoate and chlorpyrifos were used to study the distribution of pesticide residues on an apple surface by SERS microscopy imaging. Our study shows that this method can achieve rapid and quantitative detection and obtain basic information about the distribution of pesticide residues during pesticide application, which has the potential to be applied to the studies of the diffusion and absorption processes of pesticides in agricultural products.

## Abstract 2

Surface-enhanced Raman spectroscopy (SERS) is an emerging technique for the detection of pesticide residues on food surfaces, permitting quantitative measurement of pesticide residues without pretreating the sample.

## Graphical abstract

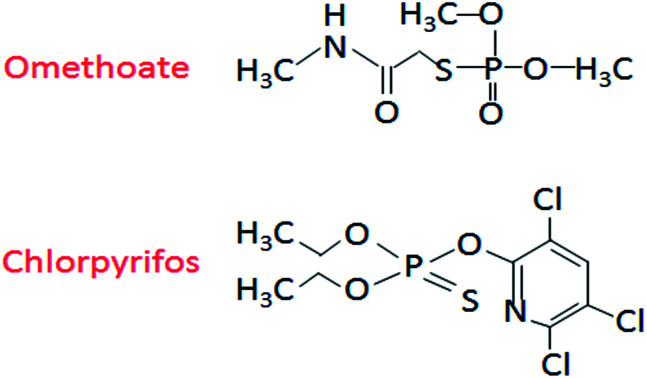

## Introduction

Omethoate and chlorpyrifos are efficient insecticides with a strong contact and stomach toxicity, and are widely used in the cultivation of fruits, vegetables and other crops. After being applied to kill pests, a portion of the pesticides remains on the surface of the crop. This residue, coupled with overuse of the chemicals on the crops, can exert a certain degree of harm to the human body.^1–3^ Fruit is necessary for people's daily life, so the detection of pesticide residues on the surface of the fruit is crucial.

Gas-phase or liquid-phase chromatography-mass spectrometry exhibits high sensitivity in pesticide analysis, but its operation is complicated and time-consuming.^4–6^ The colorimetric analysis method is faster, but is more likely to destroy the sample.^7,8^ In the field of spectroscopic methods, our research group used laser-induced breakdown spectroscopy to measure the content of pesticide residues on the fruit surface.^9,10^ Another possible method is Raman detection, but the standard Raman scattering signal is weak and cannot reach the level of detection for this application. The surface-enhanced Raman spectroscopy (SERS) technique, however, detects the molecules adsorbed in the surface of roughened metal nanomaterials (gold, silver, copper and so on) to produce physical and chemical enhancements that increase the Raman signal intensity by 10^10^ to 10^11^ times.^11,12^

At present, the SERS technique has been widely used in the detection of pesticide residues on food surfaces. Fang *et al.* detected the pesticide residues on fruits using Ag nanoparticles colloid as the enhancing substrate.^13^ Liu *et al.* measured pesticides on apples, mangos and other fruit using the shell thickness-dependent Raman enhancement method.^14^ Yang *et al.* detected pesticides on apples using silver nanoshells as the SERS substrate.^15^ Zhai *et al.* studied the content of chlorpyrifos on apple skin using gold nanoparticles as the SERS substrate.^16^ Zhang *et al.* measured multiple pesticides on an apple surface using SERS technique,^17^ while Albuquerque *et al.* detected malathion on food surfaces with the SERS method.^18^

The above research were mainly focused on the single Raman spectrum of a whole sample, but real-life applications tend to focus on the distribution of pesticides on the surface of the agricultural products to research the diffusion and absorption processes of pesticides.^19–21^ Therefore, in this study, the SERS microscopic imaging was used to explore the imaging detection of pesticide residues on the surface of fruits and leaves.

## Materials and methods

### Materials

**Fig. 1:**
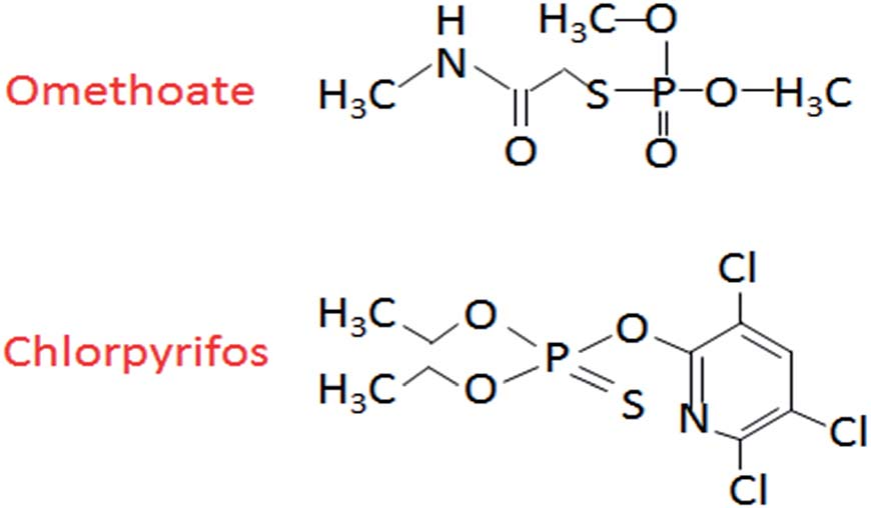
The molecular structures of omethoate and chlorpyrifos.

The apple (Red Fuji) used in this study was purchased from the Fruit Overflowing Supermarket (Haiding District, Beijing), while the omethoate (concentration 528 g L^−1^) and chlorpyrifos (concentration 480 g L^−1^) pesticides were selected from the Beijing Academy of Agriculture and Forestry Testing Center. Finally, the gold nanoparticle solution (particle size 20 nm, stored at 2–8 °C) was purchased from BBI Solutions (UK). The molecular structures of omethoate and chlorpyrifos are shown in Fig. 1.

### Experiment system

The spectral information of the samples was collected by high-resolution micro-confocal Raman spectroscopy (LabRAM HR Evolution, France). The experimental system includes a charge coupled device detector, laser (532, 633 or 785 nm wavelengths, optional), a high-precision three-dimensional platform, and an open/inverted microscope. The effective wave number range of the instrument was 50–9000 cm^−1^, with a spatial resolution of 1 μm in the horizontal and 2 μm in the vertical. The instrument was equipped with HORIBA Scientific's new spectrum analysis package, LabSpec 6, providing complete instrument operation and data processing capabilities to obtain fast and reliable results.

In this experiment, all measurements were made using a laser with an excitation wavelength of 785 nm and an output power of 80 mW. The laser was preheated for 15 min before use, with a 100× objective lens for correction and a 50× objective lens for the spectral acquisition process. Each measurement was a scanning range of 160 × 160 μm^2^, with 330 collection points and a 8 μm step size. The range of the wave selected for the study was 200–2000 cm^−1^.

### Experiment procedure

#### Quantitative detection process

First, six concentrations (0.0512–0.263 g L^−1^) of omethoate and chlorpyrifos solution were prepared, which is the typical concentration range in daily use. Next, a clean knife was used to cut about 1 cm^2^ of sample from the clean apple and leaf, upon which 2 μL of pesticide solution was dropped onto its center. After the droplets were thoroughly dried at room temperature (30 min), 2 μL of gold nanoparticles were added at the same position and allowed to sit until the droplets were dry enough to measure.

#### Imaging detection process

First, two concentrations (0.105 and 0.201 g L^−1^) of omethoate and chlorpyrifos solution were prepared, which were then sprayed evenly on the clean apple surface. The sizes of the spray droplets were on the scale of microns. We wait for a day (24 h) to measure, so that pesticides can be fully degraded on the sample surface. Then, a clean knife was used to cut about 1 cm^2^ of peel from the apple. Finally, 2 μL of gold nanoparticles were added at the center of the peel and allowed to sit until the droplets were dry enough to measure. We used the same procedure to treat leaf.

## Results and discussion

### Raman spectral characteristics of omethoate and chlorpyrifos

**Fig. 2:**
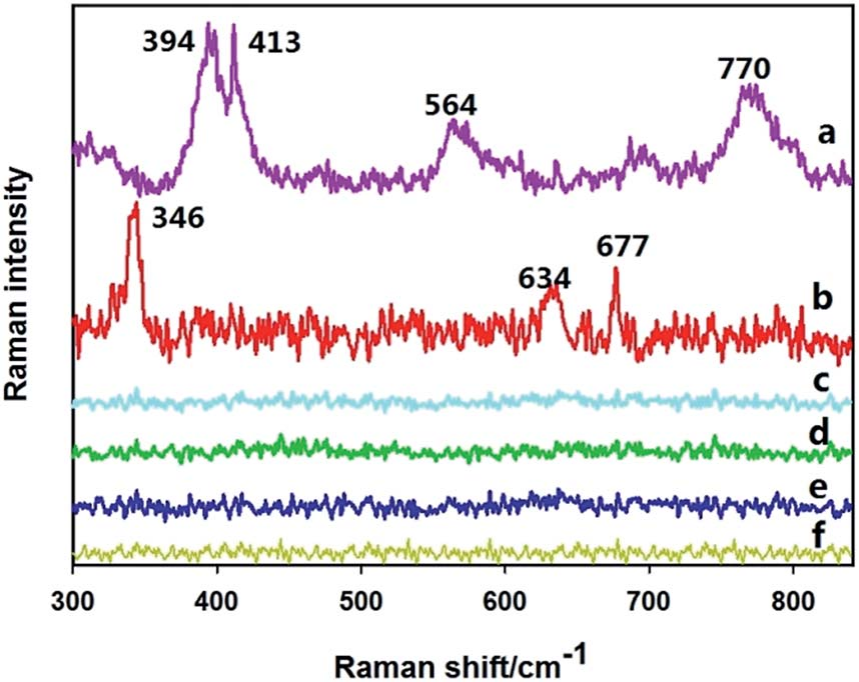
SERS spectra of an apple surface containing (a) omethoate pesticide and gold nanoparticles, (b) chlorpyrifos pesticide and gold nanoparticles, (c) omethoate pesticide only (d) chlorpyrifos pesticide only, (e) gold nanoparticles only, (f) a clean apple surface.

Fig. 2 plots the Raman spectra of various treated apple surfaces. First, a concentration of 0.105 g L^−1^ of omethoate solution was added to the surface of a clean apple, and the Raman spectrum in Fig. 2(c) was obtained, which can be compared with the Raman spectrum of the clean apple surface in Fig. 2(f). It can be seen that no obvious characteristic Raman signal is exhibited in the spectrum in Fig. 2(c). The Raman spectrum in Fig. 2(a) is of the apple surface containing 0.105 g L^−1^ omethoate and additional gold nanoparticles, where some strong Raman signals are seen to appear.

To eliminate the signal caused by the gold nanoparticles themselves, gold nanoparticles were dropped onto a clean apple surface and the Raman signal was measured (Fig. 2(e)), which does not exhibit characteristic Raman peaks. Therefore, the Raman peaks seen in Fig. 2(a) is the SERS signal of the omethoate pesticide, and the enhancement effect of the gold nanoparticles is obvious. Among the peaks, the obvious Raman peaks at 394, 413, 564 and 770 cm^−1^ are consistent with the research results of Guerrini *et al.*^22^ We also get the Raman characteristic of chlorpyrifos (346, 634, 677 cm^−1^), and can be regarded as the focus area of this study.

In this experiment, therefore, direct measurement of the areas containing pesticide residues on the apple surface did not produce detectable Raman spectra of omethoate and chlorpyrifos. However, some strong characteristic Raman signals were detected after the addition of gold nanoparticles. After eliminating interference signals from the surface of the apple and the gold nanoparticles, we obtained the Raman spectra of the omethoate and chlorpyrifos and verified the enhancement effect of the gold nanoparticles.

### SERS quantitative analysis of omethoate and chlorpyrifos pesticide

**Fig. 3:**
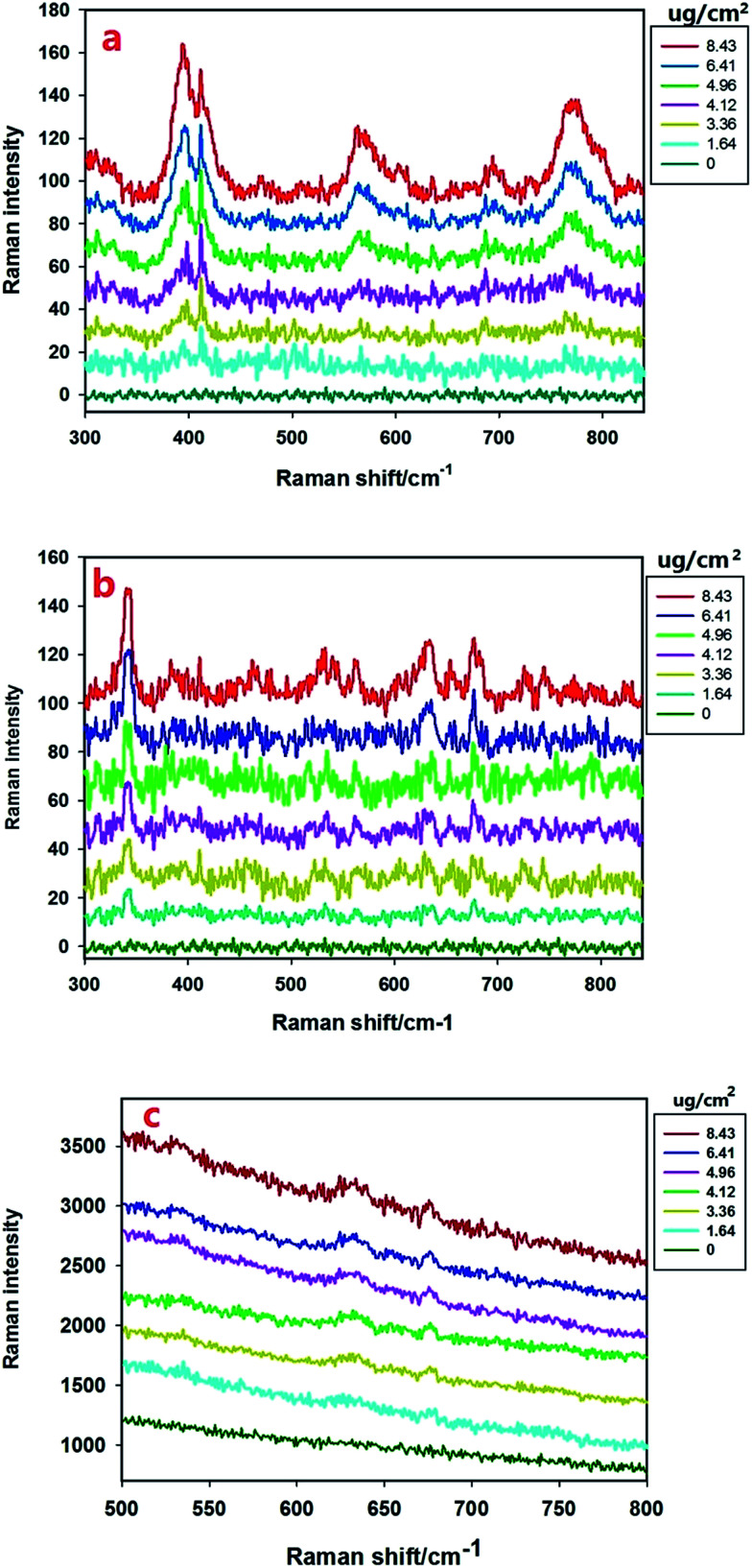
Average SERS spectra of apple surfaces (a) with different omethoate contents, (b) with different chlorpyrifos contents. Average SERS spectra of leaf surfaces (c) with different chlorpyrifos contents.

With the Raman spectra of omethoate and chlorpyrifos identified, quantitative analysis of the residue content of the pesticide was needed. To reduce the experimental error, multiple scans were obtained whose average value was used in the study, and the amounts of the reagents were strictly controlled. The SERS signals of different concentrations (0.0512–0.263 g L^−1^) of omethoate were obtained, as shown in Fig. 3(a) where the SERS intensity is seen increase with the omethoate content. A linear relationship is exhibited between the Raman peak intensity and the omethoate content at the 394, 413, 564 and 770 cm^−1^ peaks. It can also be observed from Fig. 3(a) that some Raman peaks of the characteristic band range (394, 564 and 770 cm^−1^) are shifted, which may be caused by the interaction between omethoate and the gold nanoparticles. At 413 cm^−1^, a good correlation exists between the intensity of the characteristic peak and the content without a Raman characteristic band shifted. Above all, the Raman peak intensity (413 cm^−1^) was used as the basis of the SERS quantitative analysis of the omethoate residue. We also obtained the results of the measurement of chlorpyrifos. Fig. 3(b) shows that the average SERS spectra of apple surfaces with different chlorpyrifos contents, and the Raman peak intensity (346 cm^−1^) was used as the basis of the SERS quantitative analysis. To further demonstrate the ability of SERS for the detection of pesticide on agricultural products, we also scanned a leaf with different chlorpyrifos contents. As shown in Fig. 3(c), because of the influence of fluorescence, only the 634 and 677 cm^−1^ peaks can be seen.

**Fig. 4:**
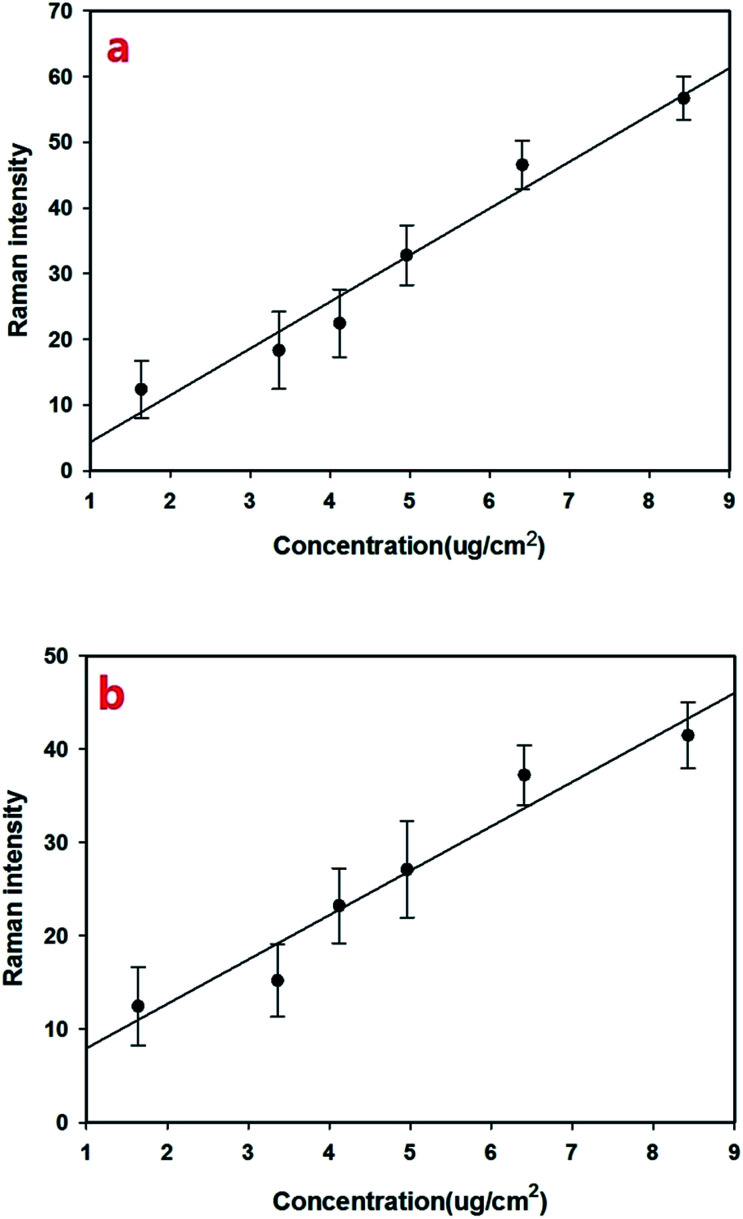
(a) Regression curve of the Raman peak (at 413 cm^−1^) intensity and omethoate content on the apple surface. (b) Regression curve of the Raman peak (at 346 cm^−1^) intensity and chlorpyrifos content on the apple surface. The error bar indicates the standard deviation of the measurement.

We then established an univariate linear regression model based on Raman shift intensities and pesticide concentrations on fruit surfaces, as shown in Fig. 4. The coefficient of determination (*R*^2^) of omethoate is 0.96 (Fig. 4a) and of chlorpyrifos is 0.94 (Fig. 4b). It is obviously that the regression curve has a high correlation that is sufficient to establish a good SERS quantitative model. The LOD (limits of detection) for omethoate and chlorpyrifos were 1.63 μg cm^−2^ and 2.64 μg cm^−2^, respectively.

### SERS imaging analysis of omethoate and chlorpyrifos residue on apple surfaces

**Fig. 5:**
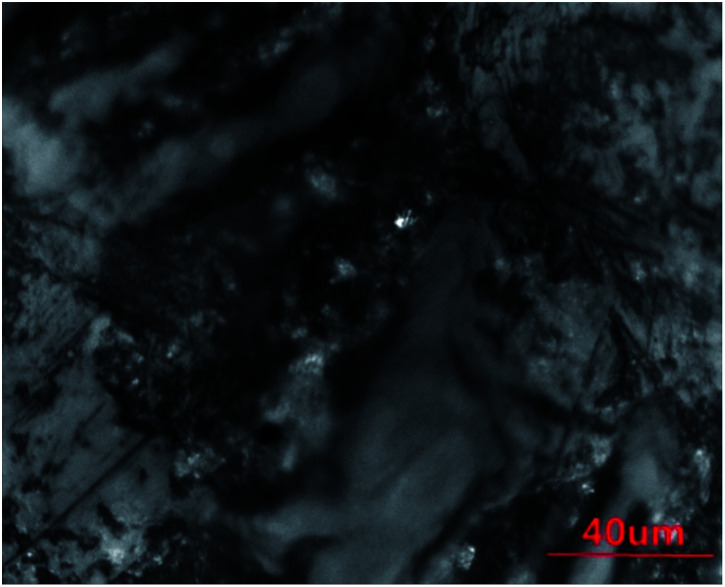
Microscope image of the apple surface.

The clean apple surface and that sprayed with the pesticide solution are objects for measurement. Fig. 5 shows the actual scanning surface area of the clean apple and leaf under the microscope, where the measurement process was performed from left to right and from top to bottom.

In the preceding section of this work, we quantified the SERS signal of omethoate and chlorpyrifos residues and established regression models. The relationship between the Raman peak intensity and pesticide content was obtained to calibrate the results of imaging analysis, whereupon the color blocks of the image represented the pesticide residue distribution and its magnitude of concentration on the apple and leaf surface.

**Fig. 6:**
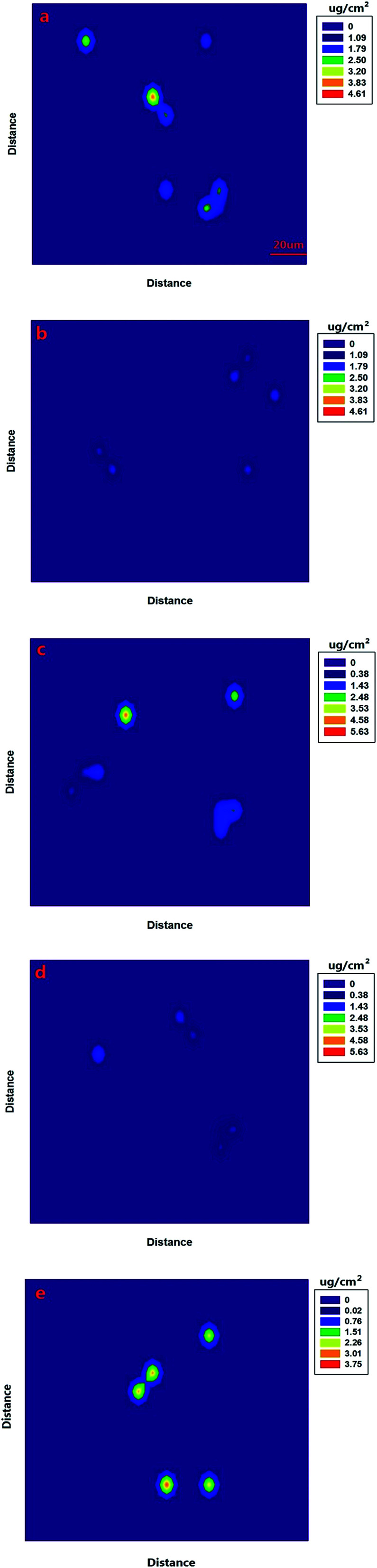
SERS imaging of the omethoate solution sprayed on the apple surface with concentration of (a) 0.201 g L^−1^ and (b) 0.105 g L^−1^. SERS imaging of the chlorpyrifos solution sprayed on the apple surface with concentration of (c) 0.201 and (d) 0.105 g L^−1^. (e) SERS imaging of the chlorpyrifos solution sprayed on the leaf surface with concentration of 0.201 g L^−1^.

Fig. 6(a) shows a SERS image of the apple surface containing the 0.201 g L^−1^ omethoate solution. First, the SERS signal of the omethoate pesticide was obtained with the experiment procedure discussed in the Materials and methods section. Then, the value of the characteristic peak intensity (413 cm^−1^) was identified at the corresponding position, exhibiting the actual distribution of omethoate residues. In the image, stronger signals are shown as a brighter color in the corresponding point. We observed that the color representing the concentration value of the image in Fig. 6(a) is lower than the omethoate concentration originally used (0.201 g L^−1^). It could be inferred that the omethoate solution was degraded, which resulted in fewer pesticide molecules remaining on the apple surface. Overall, the surface of the apple was not smooth, and the area of the pesticide residues appears irregularly shaped. Meanwhile, this irregularity also resulted in a great variation in the concentration level of some adjacent locations. Even so, we were able to clearly observe the change in concentration and the general distribution of pesticide residues on the apple surface.

We also sprayed a 0.105 g L^−1^ omethoate solution on the apple surface (Fig. 6(b)), and compared the results with those from the higher omethoate solution concentration (0.201 g L^−1^) in Fig. 6(a). It can be seen that the lower omethoate concentration produces a reduced intensity of color on the partial area and a reduced Raman signal intensity compared to the results from the higher omethoate concentration, which is consistent. Further, the omethoate concentration value calculated using the Raman spectra is also lower than the actual concentration used (0.105 g L^−1^), though the difference between the measured and actual concentrations is not as large as that found with the higher omethoate concentration (0.201 g L^−1^) in Fig. 6(a). This may be because the absorption of omethoate on the apple was more likely to reach equilibrium in a low concentration state, so that the number of pesticide molecules remaining on the apple surface was relatively more. We also obtained the SERS image of chlorpyrifos (0.201 and 0.105 g L^−1^), as the Fig. 6(c and d) shows, similar to the results of omethoate analysis. In addition, we can get the distribution of pesticide residues on the leaf surface Fig. 6(e). According to the SERS imaging analysis of omethoate and chlorpyrifos, we can more intuitively understand basic information regarding pesticide residues on an apple and leaf surface.

## Conclusions

In this study, we used the SERS technique to obtain the Raman spectra of omethoate and chlorpyrifos. Raman shift of 413 cm^−1^ (omethoate) and 346, 634 cm^−1^ (chlorpyrifos) was chosen as the peak of interest, and the regression curve model was established using six pesticide solution concentrations (0.0512–0.263 g L^−1^). Based on the quantitative analysis results, the SERS imaging of two pesticide concentrations (0.201 and 0.105 g L^−1^) on the apple and leaf surface was studied. The experiment results described the relationship existing between the Raman spectra of different pesticide concentrations and revealed basic information regarding the pesticide residues on the apple and leaf surface, which is potential for further studies of the diffusion and absorption processes of pesticides in fruits. The aim of our study was to investigate the ability of SERS for pesticide residue distribution on fruit surfaces during pesticide application, while the discussions of some important issues were not involved, such as the enhancement factors and the distribution of the hot spots. We will further study the distributions of the gold nanoparticles in SERS measurement to enhance both the sensitivity and quantitative ability of the method in future work.

## Conflicts of interest

The authors declare there is no conflicts of interest.

## Supplementary Material
